# Evaluation of Local Media Surveillance for Improved Disease Recognition and Monitoring in Global Hotspot Regions

**DOI:** 10.1371/journal.pone.0110236

**Published:** 2014-10-15

**Authors:** Jessica S. Schwind, David J. Wolking, John S. Brownstein, PREDICT Consortium, Jonna A. K. Mazet, Woutrina A. Smith

**Affiliations:** 1 One Health Institute, School of Veterinary Medicine, University of California Davis, Davis, California, United States of America; 2 Children's Hospital Informatics Program, Boston Children's Hospital, Boston, Massachusetts, United States of America; 3 Department of Pediatrics, Harvard Medical School, Children's Hospital Boston, Boston, Massachusetts, United States of America; Quensland University of Technology, Australia

## Abstract

Digital disease detection tools are technologically sophisticated, but dependent on *digital* information, which for many areas suffering from high disease burdens is simply not an option. In areas where news is often reported in local media with no digital counterpart, integration of local news information with digital surveillance systems, such as HealthMap (Boston Children’s Hospital), is critical. Little research has been published in regards to the specific contribution of local health-related articles to digital surveillance systems. In response, the USAID PREDICT project implemented a local media surveillance (LMS) pilot study in partner countries to monitor disease events reported in print media. This research assessed the potential of LMS to enhance digital surveillance reach in five low- and middle-income countries. Over 16 weeks, select surveillance system attributes of LMS, such as simplicity, flexibility, acceptability, timeliness, and stability were evaluated to identify strengths and weaknesses in the surveillance method. Findings revealed that LMS filled gaps in digital surveillance network coverage by contributing valuable localized information on disease events to the global HealthMap database. A total of 87 health events were reported through the LMS pilot in the 16-week monitoring period, including 71 unique reports not found by the HealthMap digital detection tool. Furthermore, HealthMap identified an additional 236 health events outside of LMS. It was also observed that belief in the importance of the project and proper source selection from the participants was crucial to the success of this method. The timely identification of disease outbreaks near points of emergence and the recognition of risk factors associated with disease occurrence continue to be important components of any comprehensive surveillance system for monitoring disease activity across populations. The LMS method, with its minimal resource commitment, could be one tool used to address the information gaps seen in global ‘hot spot’ regions.

## Introduction

Traditional disease surveillance systems are reliant on health data from hospital or public health department records to detect and monitor disease across populations. Recently however, public health surveillance has expanded to included digital information [1]. Digital disease surveillance involves the collection of health-related information from web-based or mobile telephone sources to better understand the distribution, incidence, or risk factors associated with disease. The major benefits of using digital disease detection methods include the rapid acquisition and dissemination of real-time or near real-time information and an ability to significantly expand the quantity of information not easily gained through more traditional methods of disease surveillance through official records [2], [3].

Initial digital disease detection and early warning systems were pioneered by a group of scientists through the Program for Monitoring Emerging Diseases (ProMED) in 1993 [4]. As one of the largest publically available disease reporting systems globally, ProMED relies on the digital transfer of disease information in real-time from participating members. By 2007, ProMED had close to 40,000 subscribers from over 165 countries and was generating seven to ten reports daily concerning global disease events [5]. Other prominent organizations utilizing digital resources for disease detection include the Public Health Agency of Canada’s Global Public Health Intelligence Network (GPHIN), World Health Organization’s Global Outbreak Alert & Response Network (GOARN), Infectious Diseases Society of America’s Emerging Infections Network (EIN), and the European Union’s MediSys. All of these epidemic intelligence networks rely largely on the transfer of digital disease information as the core of their systems. Digital disease detection tools, because of better technology and the ability to generate impact through the rapid acquisition and spread of data, are now critical to the success of any large surveillance system [6].

Concurrently, innovative approaches to quickly identify disease occurrence through non-traditional sources are being developed and evaluated. HealthMap was developed as on online tool for the visual presentation of reported disease incidence by location [7]. As a web-based surveillance tool, HealthMap aggregates multiple online data sources (e.g. GoogleNews, RSS feeds, ProMED alerts, and other online surveillance notifications) for outbreak monitoring and real-time surveillance of emerging and re-emerging health threats [8]. The use of health information technology for disease monitoring through tools such as HealthMap provides the capability to increase the quality, quantity, capacity, and timeliness of today’s global health surveillance systems.

Despite these advancements, gaps exist in disease detection through online and digital media sources [9], [10]. In areas with limited internet access and connectivity, establishing accurate measures of local disease activity through digital disease databases is difficult, yet it is often these areas that have the least capacity for disease detection, reporting, and response. In less-developed regions, health events of global importance may simply be reported in local television and radio broadcasts or recorded in local print media in local or regional languages. In countries where limited surveillance capacity is further diminished by information gaps concerning disease events, the establishment of early warning systems for disease outbreaks is particularly challenging. Given this disparity, accessing and translating local information for inclusion in global disease surveillance databases, such as HealthMap, could be an important, easily implementable, and low-cost step towards the early recognition of diseases.

In recognition of the limitations of digital methods for detecting disease events in developing areas where the potential for disease outbreaks are high, the United States Agency for International Development (USAID) Emerging Pandemic Threats PREDICT project developed a local media surveillance method and piloted its implementation. Therefore, the objective of this research was to determine if health information collected from local print media was an effective and worthwhile contribution to a digital surveillance tool like HealthMap. In this study, we report on the findings of a structured evaluation of the LMS pilot project conducted in 2012–2013, including a description of the surveillance method, an assessment of its attributes, and a determination of the value of including local media surveillance of health events within existing digital media surveillance platforms.

## Materials and Methods

### Project Description

In 2009, USAID launched the Emerging Pandemic Threats (EPT) Program in order to address the threat to human health posed by emerging infectious diseases of animal origin [11]. The EPT program is comprised of four main projects: PREDICT, PREVENT, IDENTIFY, and RESPOND, and involves other major public health partners such as the US Centers for Disease Control and Prevention (CDC), the World Health Organization (WHO), the Food and Agriculture Organization (FAO), and the World Organization for Animal Health (OIE). The PREDICT project was designed to monitor for and increase the local capacity in ‘geographic hot spots’ to identify the emergence of new infectious diseases from wildlife that could pose a major threat to human health [12]. These hotspots are areas with a history of disease emergence or are considered high-risk for spillover of zoonotic diseases from animals to people, including East and Central Africa, the Gangetic Plain of South Asia, Southeast Asia, and the Amazonian region of South America [13]. In 2010, after recognizing that disease and health alerts reported in local Kiswahili news media were not reaching global digital disease surveillance networks like HealthMap, PREDICT initiated the systematic screening of local media sources in Tanzania to identify key animal-human interfaces and regions considered high-risk for human-wildlife contact. Based on the success of this initial effort, local media surveillance was expanded to additional PREDICT countries, and we initiated a formal evaluation process.

### Implementation

**Figure 1:**
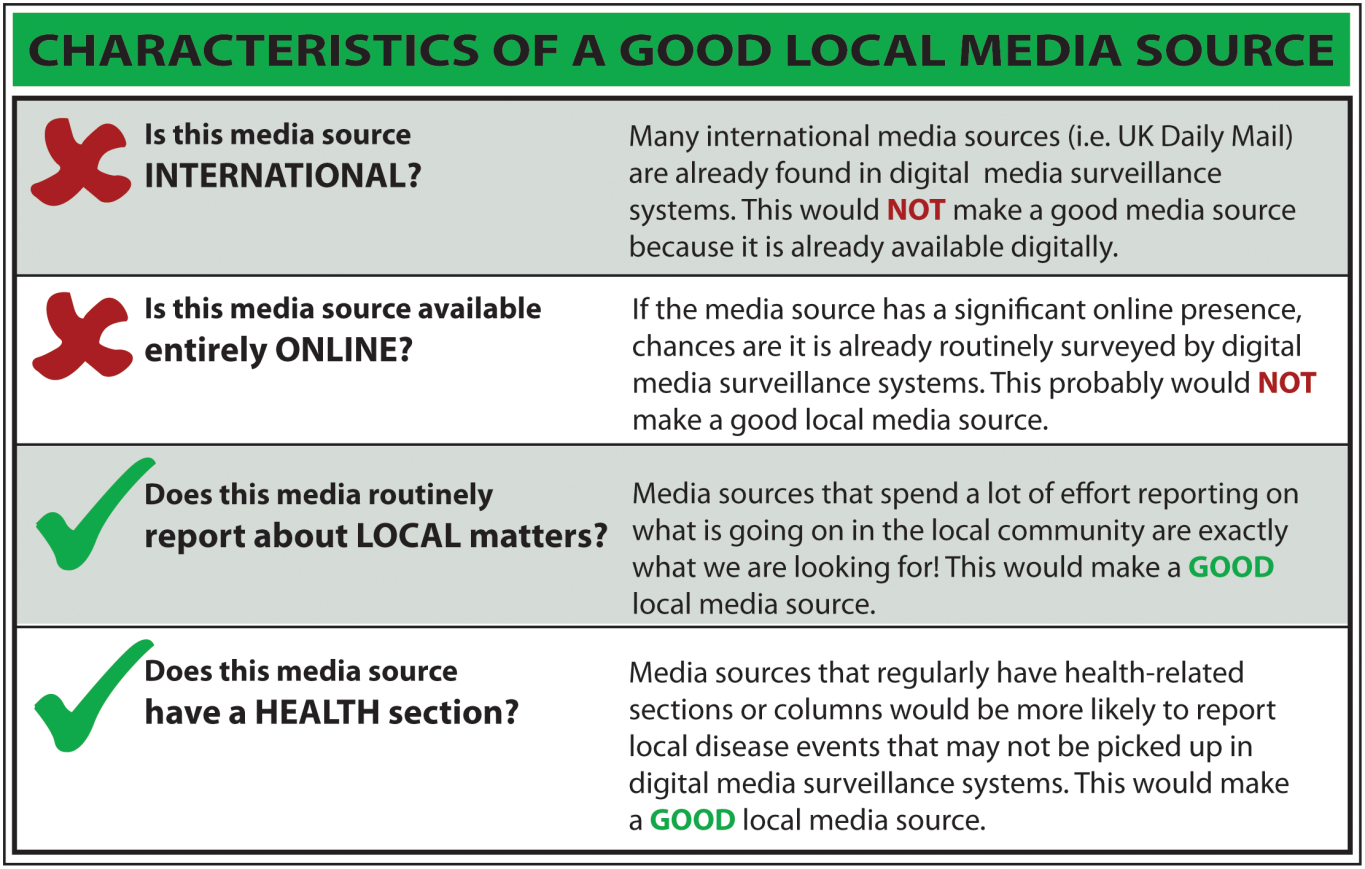
Guidelines provided for the media source selection process. These general guidelines were provided at the onset of pilot implementation in each country to help the team members select the best weekly media sources for surveillance.

**Figure 2:**
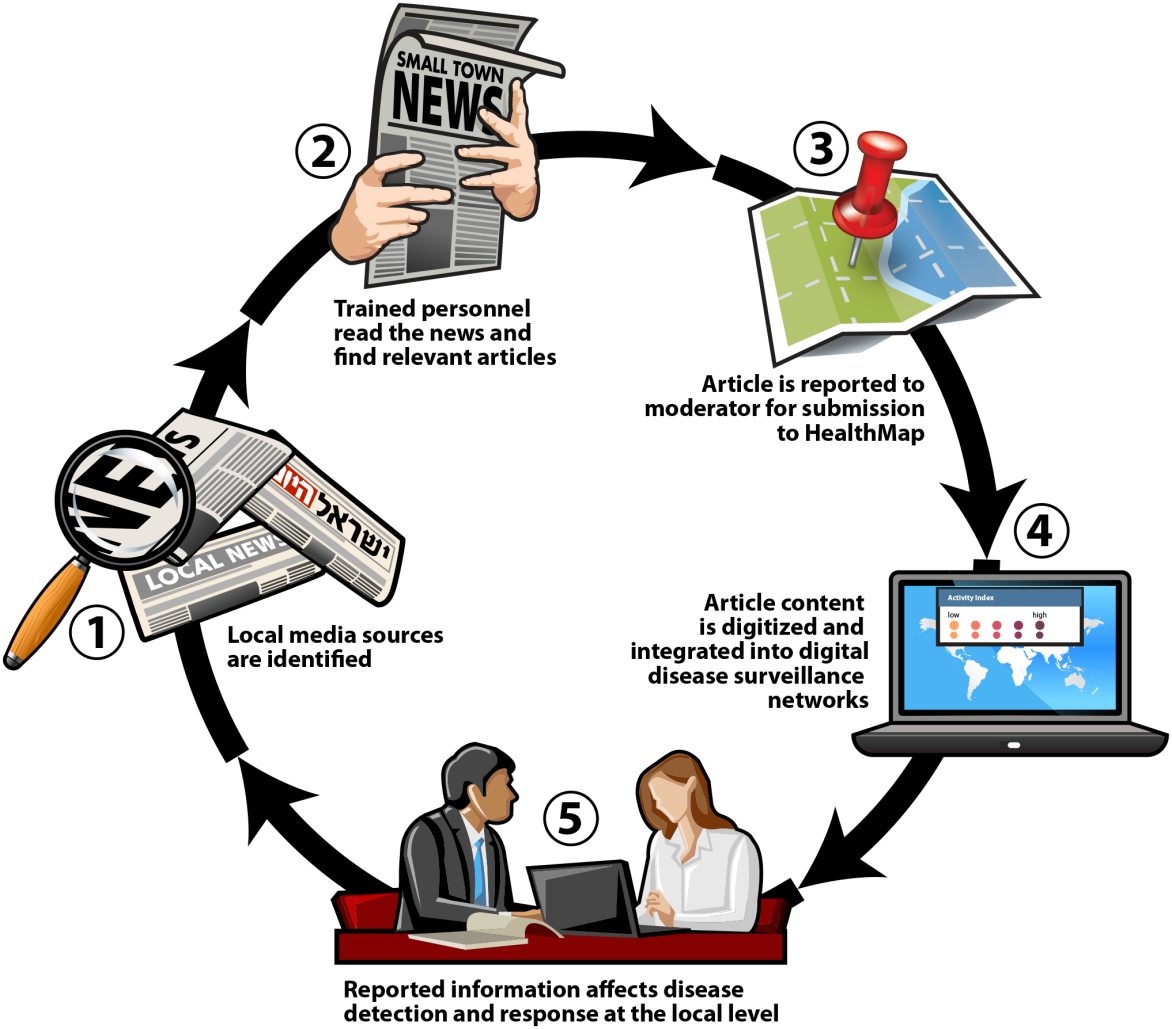
*Data and information flow through the local media surveillance pilot. From source selection at LMS implementation to the weekly reporting of local health events, the addition of offline and local information, especially from languages unsupported by digital surveillance networks, fill critical data gaps in global disease recognition and monitoring. *Striking image.

At the start, team members in each participating country were asked to perform a complete inventory of all print media available in their local area. Television and radio sources were excluded due to the difficulty of obtaining transcripts of broadcasts for reporting to HealthMap. Participating countries were encouraged to visit multiple media kiosks to ensure all print sources were documented. From this comprehensive inventory, participants (with guidance from the evaluation team) selected the sources they felt were most important and relevant to PREDICT LMS. To select and prioritize a ‘good’ media source, a source selection tool was provided (Figure 1). To focus efforts, team members reduced the number of scanned media sources to 3–6 sources per week. Selected sources were then reviewed to ensure they were not currently feeding into main news aggregation sites, including Google News or HealthMap. Participating PREDICT teams in each country were trained to screen local media for stories that may be related to a relevant health event. Over the course of the 16-week evaluation period, participants reported the time spent and sources surveyed to determine average weekly surveillance effort. If a health event was identified, participants completed a brief report form and submitted a scanned version of the original article to a PREDICT LMS moderator for monitoring and review. The review process ensured communication of all relevant information about the article and health event and supported resolution of any issues in translating the original article to English for reporting. Reports were then sent by the moderator to HealthMap for inclusion in their digital disease surveillance system. Figure 2 shows the information flow of surveillance data through LMS.

### Data Sources

Data sources for the evaluation included the initial media inventory and the weekly LMS reporting forms completed by participating PREDICT teams, HealthMap-specific data derived from sources such as online news aggregators and validated official reports, and a post-participation evaluation form completed by participating PREDICT regional leaders, country coordinators, and weekly readers. Weekly readers were employees chosen to read the selected media sources and complete the weekly reporting forms. Country coordinators oversaw the implementation of the tool in each country and ensured weekly reporting forms were accurate and complete, and regional leads for PREDICT recommended countries for participation based on feasibility and current workload. The initial media inventory collected information on the proportion of surveyed sources compared to number of media sources available in the area, as well as media source characteristics such as type (i.e. newspaper, magazine, tabloid), frequency of printing (i.e. daily, weekly, monthly, quarterly), distribution coverage (i.e. local, regional, national, international), language of source, and online URL if available. Data in the weekly LMS reporting form included name of the submitter, organization affiliation, total hours spent on LMS, and names of media sources surveyed. If an event was identified, the name, date, and source of the article; health event location; article summary; and a scanned copy were also provided. Disease or risk events with a zoonotic link were noted as they were of specific interest to the PREDICT project. To document location of the health event in HealthMap, the nearest village or town reported in the article was also included. However, in cases where participants were unable to identify the event location to the village level, a province or district was listed in the HealthMap submission. At the end of the evaluation period, HealthMap was scanned for the presence of the same event, report, or article. If the same health event was listed both by LMS and through HealthMap’s existing digital surveillance tool, the date of HealthMap publication was noted.

After the 16-week evaluation period, forms were administered to all participants to better understand the dynamics of the LMS implementation in each country. The forms specifically addressed reasons for participation, whether the team had some form of LMS active in the past, participants’ satisfaction with the current LMS method, and the feasibility of implementing LMS on a more permanent basis after the evaluation period ended. Formal ethics approval through an institutional review board was not required for this program evaluation as sources included publicly-available data on internet websites and print newspaper articles, in addition to program evaluation forms completed as a part of regular employment duties.

### Data Analysis

Raw counts were used to tabulate the number of health events reported in each country, as well as event characteristics mentioned above. For standardization purposes, a ‘health event’ was divided into two categories: ‘disease event’ or ‘risk event’, to capture actual disease occurrences along with news indicative of elevated disease transmission or amplification risk. The following definitions were provided to each team to support the selection of articles of interest:

- **Disease event**: any report containing news of an actual disease (infectious or non-infectious). For example, an article reporting an increase in the number of HIV cases, an animal die-off event, or a new case of an uncommon disease.
- **Risk event**: any report containing information on events, circumstances, or contexts that could increase potential transmission of a disease. For example, articles reporting exposure to environmental contaminants, increased interaction with wildlife, or underdeveloped sanitation infrastructure.

Weekly readers were further instructed to exclude articles on peripheral topics such as health education, health promotion, health research, or health policy. Articles reporting on health centers, distribution of vaccinations, or the implementation of new preventive health programs/policies were also not included in LMS reporting.

Reported health events from two distinct groups were compared to determine the utility of the LMS method. Weekly LMS reporting forms (group 1) were compared to global digital media reporting (HealthMap - group 2). Since the timely dissemination of data is an important component of an effective surveillance system, a health event was classified as ‘recognized’ if seen, heard, or read in a media source one calendar month before or after reporting by either the local or digital surveillance tools, or both simultaneously. A health event was classified as ‘not recognized’ if the event was not seen in one media source after it was reported in another or if it was reported, but occurred outside the one calendar month time period. In the case of a health event mentioned throughout multiple articles (e.g. during an outbreak), reported case numbers, event location, and other event characteristics (e.g. signs and symptoms of disease) were used to identify the contribution of unique information through the different surveillance strategies. Similarly, if two media sources within LMS reported the exact same content, it was only considered as one reported health event. The percentage of unique articles over the total number of submitted LMS articles was calculated in order to quantify the contribution of unique LMS data to the global digital surveillance network of HealthMap. Finally, the CDC’s guidelines for evaluating public health surveillance systems [14] were adapted to evaluate other attributes of the LMS, including usefulness, simplicity, flexibility, acceptability, representativeness, timeliness, and stability.

## Results and Discussion

Five countries began weekly surveillance on a rolling basis during a 16-week evaluation period between November 2012 and June 2013. A total of 87 (range per country: 0–29: see File S1) health events were reported through the LMS, and an average of 117 (range: 53–271) minutes were spent by each weekly reader across the 16-week evaluation period (Table 1). A total of 18 participants completed the post-participation form, including 10 weekly readers (project personnel), 5 country coordinators, and 3 regional leads for the PREDICT project. Answers from this review, as well as data gathered from HealthMap and the LMS weekly reporting forms, were utilized in order to evaluate system attributes described below.

**Table 1:** Performance characteristics of the local media surveillance (LMS) pilot by country.

Characteristics	Bangladesh	Bolivia	Cameroon	Tanzania	Uganda
# of sources identified in initial inventory	455	28	66	56	15
# of sources included in weekly surveillance	5	2	5	5	3
# of risk events* identified	6	12	25	3	0
# of disease events** identified	22	12	4	3	0
Average time (minutes) spent on LMS eachweek	271	53	137	66	60
Average # of reports per week	1.75	1.5	1.81	0.38	N/A
# of articles with zoonotic content	10	11	16	5	N/A

*Risk event: any report containing information on events, circumstances, or contexts that could increase potential transmission of a disease.

**Disease event: any report containing news of an actual disease (infectious or non-infectious).

### Usefulness

CDC guidelines define a public health surveillance system as useful if it ‘contributes to the prevention and control of adverse health-related events, including an improved understanding of the public health implications of such events’ [14]. While it is hard to prove that the recognition of a disease event through LMS made a direct impact on the control and/or prevention of a larger epidemic, early recognition of health events is a crucial component of any surveillance system. Because LMS contributed to increased knowledge of health events that otherwise would not have been reported to HealthMap, it meets the definition provided above. The specific relationship between health events reported in the LMS pilot and through HealthMap is displayed in Table 2. The findings indicated that local media surveillance contributed unique, useful, and critical information on health and disease events at the local level, potentially providing decision makers and surveillance networks with a greater amount of information regarding local disease activity.

**Table 2:** Health events identified through local media surveillance (LMS) and HealthMap’s digital disease surveillance over the 16-week evaluation period.

Source	Bangladesh	Bolivia	Cameroon	Tanzania	Uganda
LMS total	28	24	29	6	0
HealthMap total	27	106	7	6	106
LMS events only	21	15	29	6	0
HealthMap events only	20	97	7	6	106
Events seen in both LMS and HealthMap	7	9	0	0	N/A
% of unique articles found through LMS	75%	63%	100%	100%	N/A

In Tanzania, the three reported disease events through LMS were associated with suspected cases of Ebola, cholera, and caprine pleuropneumonia, while the risk events discussed slaughterhouse practices, a bat infestation, and a baboon influx, conditions associated by the LMS team in Tanzania with increased likelihood of human-animal contact and potential disease transmission. Six events were also reported on HealthMap during this same time period, but none overlapped with the events reported through LMS. The majority of events (4/6) reported on HealthMap were sourced from ProMED, indicating a limited number of external sources or data on health reporting to digital detection systems. Both the LMS and HealthMap reports covered a similar geographical area in country, with the southern region underrepresented by both surveillance methods. All chosen sources used with LMS were published in Kiswahili and had national distribution coverage.

In Cameroon, the majority of the articles reported through LMS covered risk events (25, 86%) with only 4 (14%) specifically addressing disease events. Eleven (38%) articles focused on documented instances of wildlife poaching and/or wildlife trafficking, and 3 (10%) articles focused on HIV. All HealthMap reports originated from ProMED and were concentrated around the capital, while LMS reports covered a greater area of the country. Since ProMED and HealthMap are not optimized for the reporting of risk events, we assessed the effect of the removal of risk events from the analysis on LMS and HealthMap agreement. When examining media reports on disease events only, LMS was still able to report a greater amount of health information during the evaluation period. For the LMS pilot, four out of five (80%) newspapers were published in French with the remaining one in English.

Throughout the 16-week implementation in Uganda, the team did not identify any health events through the local media surveillance efforts. Alternatively, HealthMap reported a total of 106 reports, which primarily dealt with three major outbreaks, Ebola virus disease, Marburg virus, and measles, which occurred during the evaluation period. The majority of HealthMap sources were from ProMED (61, 58%), but there were also several contributions via Google (25, 24%), RSS feeds (12, 11%), Twitter, and others (8, 7%), representing a wide variety of sources for the country. For the Ugandan LMS, two of the three selected newspapers were published in Luganda and one newspaper was published in Runyakitara.

During the LMS evaluation period in Bolivia, 24 health events were identified, 12 (50%) disease and 12 (50%) risk events. Bolivia had the greatest number of overlapping reports (9) identified through both LMS and HealthMap, but LMS still contributed a high percentage of unique articles to the HealthMap network. HealthMap covered a greater area of the country, as LMS focused on the central and northwest regions only. Several LMS articles reported disease incidents or risk factors associated with dengue (8, 33%), rabies (3, 13%), and hemorrhagic fevers (2, 8%). Of the 28 available Bolivian sources, only 2 (7%) were selected for inclusion in the local media surveillance pilot (1 national, 1 regional), as several sources already fed directly into digital media databases (Spanish is a commonly supported language of digital disease detection systems).

**Figure 3:**
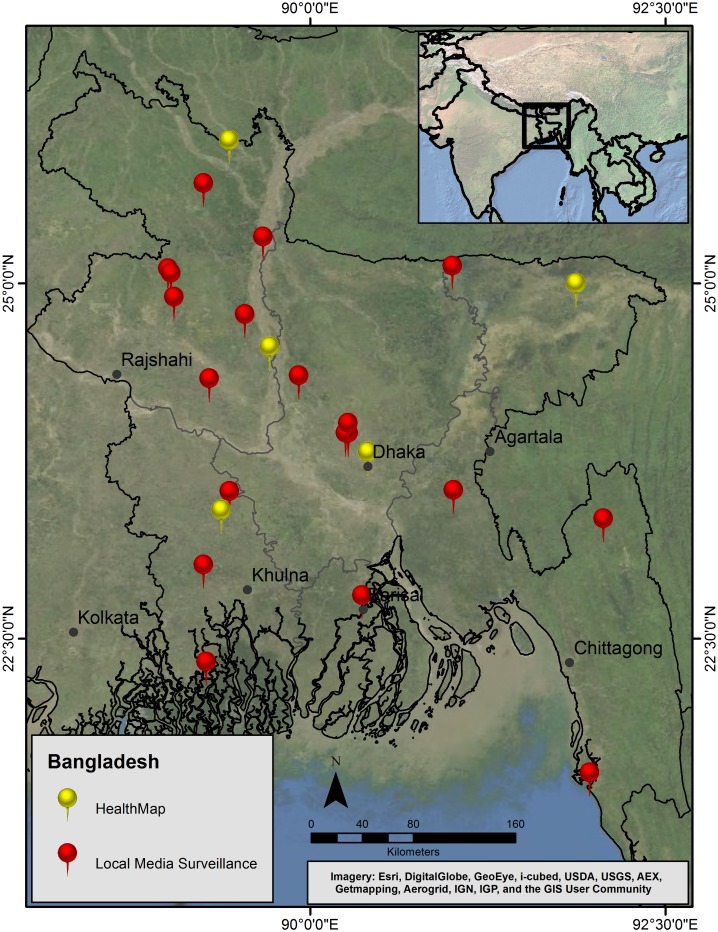
A geographic comparison of local media surveillance and HealthMap reports in Bangladesh during the evaluation period. Local media surveillance contributed to overall reporting of health events, often resulting in greater geographic surveillance coverage (Map source: ArcGIS, ESRI).

In Bangladesh, twenty-eight health events were reported through LMS, and 22 (79%) of those events were reports of disease occurrences. Major risk and disease events reported from Bangladesh included avian influenza and associated poultry outbreaks (6, 27%), diarrhea (3, 14%), tuberculosis (3, 14%), and undiagnosed syndromes (5, 23%). In a geographic comparison between results, LMS reports resulted in greater national coverage (Figure 3). In total, seven (25%) overlapping reports were made through both LMS and HealthMap. All chosen media sources for LMS were daily newspapers with regional coverage published in the Bangla language.

### Simplicity

To explore the configuration of the LMS pilot and its ease of operation, participants were asked how satisfied they were with the structure of the local media surveillance method during the post-participation form administered after the 16-week evaluation period. Sixteen out of the 18 (89%) participants indicated they were satisfied with structure, while the remaining 2 (11%) team members stated that they were neither satisfied nor dissatisfied. Country coordinators and weekly readers reported that LMS was easy to implement and participate in on a weekly basis. Several participants (6, 33%) also noted that it was helpful for identifying both disease and risk events of interest in their local area. Regarding the initial media inventory, while one team member indicated that the exercise of listing all available media sources was not useful, many others (14, 78%) understood the importance of examining the media environment as a whole for proper source selection. With a specific focus on the weekly method of reporting, 12 (67%) participants indicated that they were satisfied with the weekly reporting procedures; 5 (28%) indicated that they were neither satisfied nor dissatisfied; and 1 (5%) indicated dissatisfaction. However, the one team member expressing dissatisfaction suggested submitting reports monthly rather than weekly. Several participants (5, 28%) specifically stated that the weekly reporting provided sufficient time for media screening and reporting, given the intended objective of early warning for disease surveillance. The findings suggested that the weekly frequency of reporting was a good balance considering team members’ workloads and the need to report articles in a timely manner.

### Flexibility

Flexibility – the ability of the system to be tailored to the specific needs of each region/country – was also examined. When asked ‘How well do you feel the local media surveillance system was able to work within the unique environment of your country?’, 13 (72%) team members indicated ‘Very well’; 3 (17%) indicated ‘Not well’; and 2 (11%) indicated ‘I don’t know’. However, two limitations of the method focused on the lack of flexibility around the source selection and the reporting language. Several participants suggested including television and radio broadcasts in local media surveillance efforts, as those sources were often the perceived main supplier of local health information. Additionally, identifying weekly readers who were able to read papers in local dialects and translate the weekly summaries into English was mentioned as a difficulty for several country coordinators. Future LMS implementations should develop methods for the reporting of television- and radio-broadcasted health-related events in areas where these forms of media are predominately used to convey important information, as well as consider LMS bilingual needs when determining personnel requirements and responsibilities.

### Acceptability

We addressed the willingness and ability of personnel in PREDICT countries to participate in the LMS pilot. Of the six invited countries, five completed the 16-week evaluation period resulting in a participation rate of 83%. However, three countries indicated that they were already conducting some form of local media surveillance prior to the implementation of this pilot, though not structured or systematic. Four out of five (80%) country coordinators reported that they would be likely to continue some form of LMS after the pilot evaluation was complete. With regard to why participants were interested in permanently adopting the LMS reporting, team members stated the importance of linking local data to global databases and the usefulness of the data found for current wildlife and human disease surveillance activities. Finally, 16 (89%) participants reported that they would recommend participation in LMS to other countries if the surveillance continued.

### Timeliness

Within the context of the LMS pilot, timeliness referred to the ability of the method to identify disease events and report them to digital disease detection tools in a timeframe enabling utilization of the information by decision makers and other stakeholders. On average, weekly reports were submitted to the PREDICT moderator every Monday. Once all the information was reviewed and refined for submission, it was forwarded to HealthMap for final approval. This additional step was required before the report appeared publicly on the HealthMap interface. Delays in the reporting process from country level to moderator were generally attributed to team members working in the field, requests for additional information by the moderator, or the postponements of approvals from HealthMap post submission due to website optimizations or improvements. However, when asked if participants wanted to continue reporting to a PREDICT moderator or report directly to HealthMap using a dedicated online report form or application, 9 (50%) participants stated a preference of reporting through the moderator, so that health events could be monitored and reviewed for completeness, refined, and improved as needed before public submission.

### Stability

We assessed both the reliability (i.e. the ability to collect, manage, and provide data without failure) and availability (i.e. the ability to be operational when it is needed) of the LMS method in-country. Each country was able to contribute 16 weeks of data during the evaluation period; however, two countries reported distribution issues with a limited number of media sources. While participants completed a LMS report form every week, not all media sources were available for review on a weekly basis. In one country, team members were able to request back issues from the publisher as needed, but this was not possible in all instances. In conclusion, seventeen (94%) participants believed the local media surveillance was an important part of establishing an early warning system for emerging health threats. Diligence, commitment, and financial support for the staff to review newspapers each week and report health events were key to the success of the pilot.

Digital disease detection systems have changed the way public health information is utilized and communicated globally [15]. Through digital tools, real-time recognition and monitoring of disease events have significantly enhanced the capability of decision makers to respond to disease threats. Despite this capability, information is only valuable when it is utilized. To that end, several recommendations from leading researchers have focused on developing the in-country capacity of surveillance systems, information managers, and decision makers as crucial components of truly functional global early warning systems. [11], [13], [16] In addition to traditional health surveillance elements, part of that “capacity” includes the general infrastructure, institutions, and organizations that support the seamless migration of digital data into detection systems optimized for disease surveillance. We recognize digital disease detection systems as critical tools for epidemic intelligence and real-time disease monitoring, but also acknowledge their limitations in integrating information from less-developed areas of the world, areas like the Congo Basin tropical forests – areas on the edge of agricultural intensification where disease emergence poses significant risk, and perhaps most importantly areas where disease surveillance is simply not feasible. Monitoring predominant forms of media in local dialects and systematically integrating these local media sources into digital disease detection systems can help close these data gaps, enabling decision makers to better monitor disease conditions in remote areas and support planning and control efforts.

Our evaluation showed that surveillance of local media adds value to global digital disease detection systems for public health surveillance. Through the routine monitoring of local print newspapers, we found that the majority of health events reported locally through LMS were not captured by HealthMap’s digital aggregation algorithms (71 out of 87 articles), because these reports were not available digitally. The strength of the LMS was its ease of implementation. LMS was not resource-intensive, requiring minimal personnel and financial commitment. In Tanzania, for example, the total cost for newspaper surveillance during the 16-week evaluation period was $62.33 with an average of 60 minutes spent screening media in personnel time. As media source selection is refined and focused in each country, the cost-benefit ratio of the method improves. Because HealthMap does not currently support all languages, LMS participants captured local information and knowledge and communicated it to a global audience. This in turn contributed to the overall acceptance and perceived value of the system in each participating country. By integrating LMS with existing digital detection systems like HealthMap, we extended the reach of both surveillance methods, introducing local media and disease events to a global audience, while indoctrinating participating local teams with the value and benefits of digital disease detection systems and tools.

Despite the demonstrated benefits of the LMS pilot, our evaluation identified limitations and areas for improvement required for LMS to reach its full potential as a tool for global disease surveillance and epidemic intelligence. No attempt was made to verify the accuracy of the details reported through the local media. As such, caution must be given when trying to interpret the findings from all surveillance systems that utilize non-traditional methods for data, such as newspapers and search engine queries. Additionally, the variation of results across countries highlighted that our approach was extremely reliant on proper source selection. In some countries, the limited number of articles may have been attributed to a source selection bias, where teams selected newspapers that were unlikely to report health events. Specifically in Uganda, team members stressed many of the national newspapers were also available digitally, a disqualification criteria in our source selection guidelines, and a factor potentially related to the location of our Uganda team in Kampala, Uganda’s commercial and political center. However, as demonstrated by results from Bolivia, it was clear that availability of digital news editions should not be the sole limiting factor in the source selection process, as not all digital sources feed directly into news aggregation sites. In other cases, possibly due to cultural or logistical reasons, health events may simply just not be reported in the local media, as evidenced by a recent study reporting similar findings even in the more established Canadian media environment [17]. As noted by several of the participants, LMS should not be restricted to print media. An online mechanism for reporting stories heard/seen through other media sources, like television or radio, should also be developed and evaluated. With the inclusion of television and radio reports in LMS, greater programmatic benefits are likely to be seen due to the addition of unique information included in HealthMap’s digital surveillance network. In contrast, countries where media is regulated or controlled will limit the ability for LMS to contribute to the overall knowledge of current health events in digital surveillance networks.

Several teams reported newspaper distribution issues in their area; a lack of reliability around the media source acquisition harms the systematic nature of data collection that is important to a sustainable surveillance system. One potential solution in the implementation of future LMS programs would be to hire additional qualified readers to review more sources for greater, more reliable, coverage of all relevant media, or to integrate readers at the source of publication or in the news room, where reports on health events could be fed directly into digital detection systems more rapidly, bypassing print and distribution delays. To foster greater LMS use and adoptability across global regions, additional areas of improvement should include the development of site-specific goals and objectives in order to best utilize findings from LMS. Finally, with regard to LMS performance, considerable gains may still be made in improving timeliness of reporting as any step in a surveillance system that requires human action has the potential to slow down the reporting process. With a weekly reporting time frame and delays in pushing submitted reports to the public interface on HealthMap, upwards of 7+ days were average before LMS data could be viewed by stakeholders and decision makers. If LMS was adopted and scaled up in multiple locations across the globe, different options exist for improving this timeline, including direct posting to HealthMap using existing online applications (OutbreaksNearMe) or a restructuring of the reporter-moderator-HealthMap integration process.

Unanticipated benefits were realized over the course of the evaluation. For example, teams reported that the articles found through LMS helped identify high-risk animal-human interfaces for zoonotic disease transmission and guided field investigations for PREDICT’s wildlife disease surveillance activities. While the PREDICT project’s charge through USAID was to monitor for and increase the local surveillance capacity in hotspots in order to identify the emergence of potentially zoonotic pathogens in high-risk wildlife that could pose a major threat to human health, LMS can be tailored to each countries’ specific needs and thereby focus on other health-related events of interest (i.e. environmental exposures, traffic accidents). As more countries adopt the surveillance method and more sources are included in surveillance, other benefits of LMS could be seen. Additionally, the possibility of educating media sources and organizations in hotspot areas on where and how to best cover health-related events, along with the benefits of channeling information on those events to global disease detection systems, is significant.

This LMS method evaluation was a first step in assessing the potential benefits of linking local information with digital disease detection systems. Additional research is needed to examine differences in local and digital media content during outbreaks to better understand both the quantity and quality of media reporting in disasters, along with additional potential gaps and weaknesses in online content. Prospective research comparing LMS to digital disease aggregation sites should be conducted to further assess and quantify the timeliness of reporting and the possibility of LMS to contribute to disease early warning. Finally, more research remains to assess the sustainability of LMS in the absence of large donor funded initiatives like PREDICT and how best to integrate activities like LMS in existing national level health surveillance systems promoted and supported in the public sphere.

In conclusion, LMS contributed valuable information to the currently available global digital disease detection data. Local media surveillance provided a broader range of coverage, as well an alternate pathway for reporting health events to HealthMap. The LMS evaluation demonstrated that screening local media for health information can be an effective and worthwhile addition to active digital surveillance networks, even in areas with relatively robust internet connectivity and an abundance of online digital media. Therefore, adoption of local media surveillance should be encouraged in areas with less-developed capacity for disease detection and response.

## Supporting Information

File S1 **DOI for articles.**
(XLSX)
